# Stocking density in intensive housing and the implications for beef cattle behavior, stress physiology, and liveweight

**DOI:** 10.1093/jas/skaf034

**Published:** 2025-02-07

**Authors:** Bonnie T Mayes, Sharon G Dundon, Frances C Cowley, Lee-Emma K Norman, John M Morton, L Amy Tait

**Affiliations:** School of Environmental and Rural Science, University of New England, Armidale, NSW 2351Australia; Meat & Livestock Australia, Animal Wellbeing, Armidale, NSW 2351, Australia; School of Environmental and Rural Science, University of New England, Armidale, NSW 2351Australia; School of Environmental and Rural Science, University of New England, Armidale, NSW 2351Australia; Jemora, East Geelong, VIC 3219, Australia; School of Environmental and Rural Science, University of New England, Armidale, NSW 2351Australia

**Keywords:** allometry, cattle welfare, live export

## Abstract

Stocking density can potentially impact cattle welfare during livestock export voyages. The aim of this study was to assess selected measures that reflect the welfare of cattle housed at 3 allometric stocking densities (*k* = 0.027, 0.030, 0.047). *Bos indicus* cross *Bos taurus* steers were housed in 12 pens, each with 5 steers, for 10 d. Scan sampling of standing and lying behaviors were conducted on days 2, 5, 7, and 9, at hourly intervals. Continuous observations were conducted on the same days between 1030 and 1130 h, to count aggressive interactions. Liveweights were recorded at the start of the study, and on days 6 and 10. For a subset of focal steers (3 per pen), white blood cell counts, and fecal glucocorticoid metabolite (FGCM) concentrations were assessed on days 0, 6, and 10. More pen space led to a small increase in the number of steers lying, as well as a small increase in lying synchronicity. Results also indicated that the number of cattle lying in isolation from conspecifics is higher when more space is available. More pen space also resulted in more steers lying with outstretched legs on days 2 and 5, but there was no evidence of this after day 5. Stocking density had no important effect on day 6 or 10 liveweights or FGCM concentrations. Only small decreases in total white blood cell and lymphocyte counts between days 6 and 10 were observed, as well as small increases in neutrophil counts and the neutrophil-to-lymphocyte ratio, but all mean counts still fell within reference intervals for healthy cattle. The lack of important effects on stress physiology and liveweight suggests that the cost of attempting to adapt to pen space restriction was relatively low, leading to behavioral responses only. Results for lying behaviors also suggest that additional pen space may facilitate adaptation upon introduction to a new housing environment and is beneficial in facilitating the expression of some lying behaviors. While designed to emulate stocking densities applicable to Australian cattle export voyages, other environmental factors that may induce stress during these voyages were not present, and so the conclusions must be interpreted in the context of the controlled experimental conditions.

## Teaser

For cattle housed intensively, stocking density did affect lying behaviors but had limited important effects on stress physiology and biological fitness in the highly controlled experimental environment.

## Introduction

Australia is a major exporter of live animals and over 370,000 beef cattle were exported in 2022 (LiveCorp, 2022), with most animals exported to countries of South-East Asia (SE Asia; Phillips, 2022). For example, approximately 300,000 head of beef cattle were exported to Indonesia in 2022 (LiveCorp, 2022), departing from northern ports of Australia and lasting 6 d in duration (Phillips and Petherick, 2015). A smaller proportion of beef cattle were exported to Vietnam (LiveCorp, 2022), with a slightly longer voyage length of around 11 d. Voyages less than 10 d duration are considered “short haul” voyages in the context of Australia’s livestock export industry, and those lasting 10 d or more, but less than 31 d, are termed “long-haul voyages” (Department of Agriculture, Fisheries and Forestry, 2024). While voyages to SE Asia are relatively short compared to those traveling to the Middle East (i.e., up to 21 d; Collins et al., 2018) or “extended long-haul” voyages lasting more than 31 d (Department of Agriculture, Fisheries and Forestry, 2024), the sea transport journey still exposes cattle to complex environments with many factors that may affect their welfare.

The term welfare can be interpreted in many ways. In the context of this study it refers to the state of an animal “as regards its attempts to cope with its environment” (Broom, 1986), which indicates that welfare varies over a range from very good to very poor (Broom, 2017). If coping is achieved with little expenditure of resources or effort, welfare is likely satisfactory, but if an animal is unable to cope at all or can only do so by expending much time and energy, welfare is poor (Broom, 1986). For many stressors, behavioral responses represent the first and most biologically economic response which may allow animals to cope (Moberg, 2000), while altered biological fitness (e.g., growth) indicates that the cost of a stressor is high enough to be associated with impaired welfare and distress (Langford, 2006), or that an animal is failing to cope (Broom, 1991). Pen space allowance (area per animal) may be one of the most important factors influencing animal welfare during transportation, due to the potential effects of space allowance on the ability of animals to access resources in their environment, and to engage in or avoid social interactions (Lee et al., 2012). In addition, other authors have suggested that space allowance in intensive housing environments impacts an animal’s ability to rest and be comfortable, their ability to thermally regulate, and their ability to move around freely and perform certain behaviors as they desire (Hurnik and Lewis, 1991; Petherick and Phillips, 2009; Richmond et al., 2017; Dunston-Clarke et al., 2020). As for other challenges, how well an animal adapts to an intensive housing environment in which pen space is restricted will be reflected in the normality of its biological functioning (e.g., behavior, neuroendocrinology, immune competence, metabolism; Moberg, 2000; Hemsworth et al., 2015). Despite the effects which have been identified for stocking density in intensive housing environments to date, more research on the space allowances which are specific to livestock export voyages is needed.

Stocking density requirements during livestock export voyages are governed by the Department of Agriculture, Fisheries and Forestry, in the Australian Standards for the Export of Livestock (Department of Agriculture, Fisheries and Forestry, 2024). Since 2018, stocking densities during Australian livestock export voyages have been determined using allometric principles (Australian Livestock Exporters’ Council, 2018). These principles describe relationships among physical measurements of an object and changes in the size or volume of the object, so these measurements can be used to allocate space over a range of liveweights (Gonyou et al., 2006). The allometric equation used to calculate space allowances (Petherick and Phillips, 2009) is:


$$\begin{array}{l} Area\;per\;animal\;(m^{2})\;=\;\text{k}W^{0.66} \end{array}$$

In this equation, W is the liveweight of an animal (W) in kg and *k* represents a space allowance coefficient constant.

Currently, cattle exported from a northern port of Australia to Indonesia or Vietnam are stocked at an allometric *k* value of 0.030, or 0.026 if further approval has been provided by the Department of Agriculture, Fisheries and Forestry (Department of Agriculture, Fisheries and Forestry, 2024). Previous authors have suggested that a *k* value of 0.027 is sufficient for allowing all animals to lie simultaneously, but that current recommendations for space allowances for sheep, which are stocked at a similar density (*k* = 0.030; Department of Agriculture, Fisheries and Forestry, 2024), may be inadequate for allowing lying in preferred positions (Mayes et al., 2022) and may also promote pushing and aggression (Navarro et al., 2018). Furthermore, Petherick and Phillips (2009) state that a *k* value of 0.047 is required for livestock to transition between standing and lying, based on behavioral and kinematic observations performed in cattle (Ceballos et al., 2004). While there has been one study into the effects of stocking density on cattle housed intensively during livestock export voyages (Ferguson and Lea, 2013), the authors were not able to assess a broad range of validated welfare indicators and instead relied on measures of productivity and limited behavioral outcomes. As such, there is a need for more empirical evidence on the effects of space allowances on cattle for periods of time which are comparable to livestock export voyages. Research should broadly assess the biological functioning of cattle, including behavior, stress physiology, and liveweight (as an indicator of metabolism or biological fitness; Broom, 1991) to provide an indication of the biological cost of coping with restricted pen space.

The aim of this study was to assess selected measures that reflect the welfare of cattle housed at 3 stocking densities, varied by the *k* value in the allometric space equation area = *k*W^0.66^. We expected that reducing space allowance would lead to reduced cattle welfare, as indicated by assessments of behavior, stress physiology, and liveweight.

## Materials and Methods

The experiment was undertaken in a fully enclosed building at the Queensland Animal Science Precinct (QASP), Gatton, QLD, Australia. The conduct of the experiment was approved by The University of Queensland Animal Ethics Committee under the Animal Care and Protection Act, 2001 (approval ARA 2022/AE000062).

### Experimental design

The experimental design was a randomized block experiment with 3 pen-level *k* value stocking density treatments (0.027, 0.030, 0.047), using the allometric space equation: area = *k*W^0.66^. Given the liveweights of the study animals, these *k* values provided, on average, approximately 1.20, 1.33, and 2.09 m^2^ of pen space per steer, respectively. The experiment was performed in one time period, with 12 pens (4 pen replicates at each stocking density), each with 5 steers. The experiment consisted of a 7 d adaptation period, followed by a 10 d period of being housed at the allometric stocking densities. The 10 d period is comparable to relevant livestock export voyage lengths traveling from northern Australia to Vietnam. Additional physiological samples were collected on day 6, to reflect a relevant voyage length for cattle traveling from northern Australia to Indonesia.

### Animal induction and adaptation

Seventy *Bos indicus* cross *Bos taurus* steers (initial mean liveweight ± SD; 314.3 ± 23.4 kg) approximately 18 mo of age were trucked for 11 h to the experiment location, from one property located in Central Western Queensland, which is a supplier of this class of cattle for export from northern Australia to SE Asia. Prior to inclusion in the experiment, steers had been extensively managed for the duration of their lifetime up to the date of transport to the research facility.

On arrival, the steers were unloaded and split into 2 groups of 24 and 1 group of 22 and moved into 3 feedlot pens (12 × 27 m) where they were housed overnight. Each feedlot pen contained an automatic water trough, and 12 m of feed bunk length containing half their daily ration of 2% LW as 80% oaten chaff and 20% commercial shipper pellets (9.5 MJ of ME, and 12.1% CP per kg of DM; Macco Feeds, Williams, Western Australia). During the induction process the day after arrival, steers were weighed, body condition scored, and given a pour-on anthelmintic (CattleGuard, Zoetis Australia Pty Ltd, Rhodes NSW Australia). The steers had their existing ear tag removed, and this was replaced with an experimental ear tag so that each steer was identifiable by a numeric value between 1 and 70. This identifying number was also marked along their sideline with StockMark (Dy-Mark, Darra, QLD, Australia). Steers also had their temperament assessed by one observer (AT) for the first 30 s of them entering the crush (i.e., a crush score) during induction, according to the description outlined in Supplementary Table 1 (a method adapted from that described by Grandin (1993)).

Following induction, the steers were moved back into their feedlot pens to begin the 7-d adaptation period, in which the percentage of commercial shipper pellets in their diet was increased over time. The steers were fed a ration based on the as-fed weight of 50% oaten chaff and 50% pellets for the first day of adaptation. The amount of chaff was reduced to 25% and 15% for days 2 to 3, and 4, respectively. From day 5 of adaptation onwards, and during the experimental period, pellets made up 100% of the ration required to maintain liveweight, with chaff also provided equating to 1% of the as-fed weight of the total daily pellets. The feed allowance for the entirety of the trial was calculated on an as-fed basis at 2% of liveweight, offered in 2 meals per day, at 0730 and 1430 h.

### Treatment allocation

During induction, 5 of the 70 steers were excluded from the study due to receiving a crush score of 4 or 5 during the induction temperament assessment. One further steer was removed due to a leg injury, and 4 more steers were excluded to normalize the liveweight distribution. The remaining 60 steers were each randomly allocated to 1 of the 12 groups, each containing 5 steers. Each group was then randomly allocated using a random number generator, to 1 of the 3 treatment stocking densities (*k* = 0.027, 0.030, 0.047). Three steers from each pen (i.e., 12 per treatment) were randomly selected as focal steers.

### Treatment pen design

On the afternoon of day 7 of adaptation, the steers were drafted into their 12 groups and moved into their indoor treatment pens, and spare steers were drafted back to one outdoor feedlot pen. The treatment pens were in a fully enclosed shed with solid concrete flooring covered by black rubber mats designed to prevent cattle from slipping. The width of each pen was 2.1 m, and the pen area was adjusted by adjusting the length to maintain allocated space within ± 0.001 m^2^ of the area required by the allometric equation, area = *k*W^0.66^ (Petherick and Phillips, 2009), where *k* is the treatment *k* value and W is the mean liveweight of the 5 animals in the pen, as measured on the morning of day 7 of adaptation. This area was then multiplied by 5 to correspond to 5 head in each pen. Each treatment stocking density was represented once within each of the 4 rows of pens, and the location of each treatment *k* value within each row was randomly allocated. Each pen contained 2 stainless steel automatic water troughs and 40 cm per steer of linear feed trough space on the outside of the pen (steers put their head through a gap in the rail to access the feed similar to on a livestock export vessel). No natural lighting was available in the facility and steers were exposed to artificial lighting between 0700 and 1530 h.

### Environmental monitoring

Two Kestrel D3 Fire Drops (Nielsen-Kellerman, Pennsylvania, USA) were suspended from the ceiling in opposite corners of the shed, continuously logged temperature and humidity data at 10-min intervals. The average dry bulb temperature (T_DB_) over all timepoints over 10 d was 21.0 °C, and the average relative humidity over all timepoints over 10 d was 77.8%. The average wet-bulb temperature (T_WB_) over all timepoints over 10 d was 18.2 °C. Supplementary Table 2 summarizes the mean T_DB_, RH, and T_WB_ on each day of the study.

### Animal management

During the experimental periods, handlers entered the shed twice per day (0700 to 0830 h and 1430 to 1530 h) for health and welfare checks, cleaning, and feeding. The health and welfare of all steers were checked first, using the Gunson inspection method (Jubb and Perkins, 2019), and then water troughs were cleaned as required. The pens were washed out using a high-pressure hose; care was taken to not wet the steers or concrete floors around the pens. At the conclusion of hosing, all feed troughs were cleaned. Pens were then fed in a random order. Feed refusals were minimal throughout the entire duration of the trial and consisted only of pellet fines. The process was the same in the afternoon, except that pens were not hosed. At all times outside of these occurrences, the shed was closed (with clean air ventilated throughout the shed) and steers were left entirely undisturbed.

### Behavior assessments

Video footage was recorded by fixed infra-red cameras (MR6822E2 and LR832, Lilin Australia Pty Ltd, Lidcombe NSW) positioned above each individual experimental pen for the entire duration of the 10 d experimental period. Cameras were positioned so that the entire pen and all animals were visible throughout the study. Behaviors were assessed after the experimental period by one observer (SD) using a scan-sampling approach for standing and lying behaviors, and another observer (LN) using a continuous observation approach for aggressive social interactions.

All steers in each pen had their standing or lying position recorded (Table 1) in a scan-sampling approach, at hourly intervals (using still images) for a 24-h period on days 2, 5, 7, and 9. Five timepoints were excluded on each day, when the steers were disturbed due to human presence (0700, 0800, 0900, 1400, and 1500 h). Hourly timepoints were selected based on previous work in sheep (Mayes et al., 2022) which found a 60-min scan sampling interval used to sample similar behaviors to those in Table 1 provided an accurate representation of behaviors compared to more frequent intervals (15, 30, and 45 min). Scan sampling behavior data were collected in Microsoft Excel (2016).

**Table 1.** Ethogram utilized to record positional behaviors for all steers at scan sampling observation times

Position	Definition
Standing	Steer is upright with at least 3 hooves (so could be performing locomotion) in contact with the pen floor
Lying 0	All 4 legs are kept close to the body. There is no gap between the lower portion of a back leg and the body. No front legs are stretched out
Lying 1	1 leg outstretched from body. There is a visible gap between the body and the lower portion of the outstretched leg.
Lying 2	2 legs outstretched from body. There is a visible gap between the body and the lower portion of the outstretched legs.
Lying 3	3 legs outstretched from body. There is a visible gap between the body and the lower portion of the outstretched legs
Lying 4	4 legs outstretched from body. There is a visible gap between the body and the lower portion of the outstretched legs
Lying unknown	Cannot see enough legs to determine position
Body 1	Steer body or limb in direct contact with another steer
Body 2	Steer is not touching another steer with its body or limb
Body unknown	Cannot determine if steer is touching another steer
Head 1	Head is held up
Head 2	Head is down and resting, placed on the floor or the pen
Head 3	Head is down and resting, placed on itself
Head 4	Head is down and resting, placed on another steer
Head unknown	Cannot determine the head position of the steer

At each timepoint, each steer was classified as one standing or lying 0 to 4 or unknown, and those lying were also classified on body contact with other steers, and on head position.

One observer (SD) was trained by BM to perform the scan sampling analysis. Training consisted of both observers applying the ethogram (Table 1) to approximately 20 pen-timepoint combinations until SD was confident in applying it independently. At the conclusion of data collection, interobserver agreement was assessed by calculating Lin’s concordance correlation and intraclass correlation coefficients for agreement in counts for the trainer (BM) and observer (SD), among 76 pen-timepoint combinations, which included data from all treatment stocking densities and all behavior observation days. The agreement was not assessed for Lying 3 or Lying 4 due to the rarity of these behaviors in the test data. SD maintained a high ability to score behaviors according to the initial training over time, with interobserver agreement for most behaviors exceeding 0.7 for both measures at the conclusion of the data collection period (Supplementary Table 3; Martin and Bateson, 2007).

Continuous behavior observations were conducted for all steers in each pen for one 60-min period on each of days 2, 5,7, and 9, from 1030 to 1130 h. Aggressive social interactions were recorded; these behaviors were recorded as point occurrences due to their short duration of less than 2 s and were defined as a steer making direct contact with a conspecific by head-butting, mounting, or pawing. Each contact was recorded as an individual occurrence. Continuous behaviors were recorded in Behavioral Observation Research Interactive Software (BORIS v. 7.13.8). The 1030 to 1130 h observation period was chosen to represent a time at which the lights were on, and steers were standing and active, as determined in a preliminary assessment of the video footage, to detect a reasonable number of agonistic interactions.

### Physiological sampling

**Figure 1.**
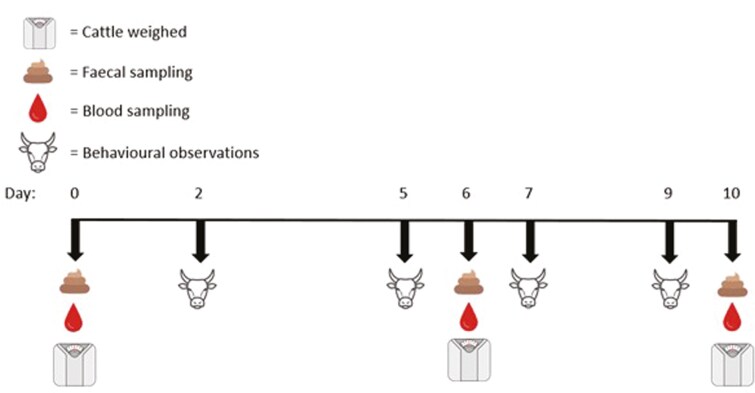
Experimental timeline depicting the days on which liveweights, blood, feces, whole of pen scan sampling behaviors, and continuous behaviors were sampled from focal steers. The 7-d adaptation period ended on day 0 and the experimental period was from days 1 to 10.

All steers were brought out of their pens and held in a crush to be weighed on the morning of day 0 (i.e., the 7th day of the adaptation period), days 6 and 10; at each weighing, steers had not been fed within the previous 12 h. The physiological samples (blood and feces) were also collected from focal steers on these days, while steers were in the crush. Blood samples were collected from focal steers to measure white blood cell counts (Figure 1). Blood samples were taken via jugular venipuncture and collected into 10 mL K_2_-EDTA vacutainer tubes (BD, Lane Cove, NSW, Australia). Blood samples were stored at 4 °C for approximately 1 h post collection, then analyzed on a Sysmex XN-1000 (Sysmex, Macquarie Park, Australia) hematology analyzer and manual cell differentiation was performed.

Fecal samples were collected from focal steers on days 0, 6, and 10 (Figure 1) while they were in the crush. A gloved hand was inserted directly into the rectum of the focal steers to collect at least 50 g of feces, which was immediately placed in a sample pot on ice. Fecal samples were frozen within 40 min after collection and stored at −20 °C until processing. Samples were oven-dried at 60 °C for 48 h and finely ground using an electric grinder. The individual samples were then analyzed using a method previously established for cattle (Campbell et al., 2019). Briefly, 100 mg of dried sample were reconstituted in 300 µL of double distilled water followed by mixing with a vortex for 5-min. This was added to 2,700 mL of 100% ethanol, vortexed for 10-min, and then spun at 2,000 × *g* for 10-min; the supernatant was decanted into glass tubes. Pellets were extracted again with 3 mL of 100% ethanol, spun at 2,000 × *g* for 10-min, and the supernatant was added to the previous extract. The extracts were dried under airflow for 5 to 6 h and then reconstituted in 500 µL of phosphate buffer saline (pH 7.4), vortexed for 10 min, and spun at 1,000 × *g* for 2 min. Concentrations of fecal glucocorticoid metabolites (FGCM) in the extract were measured in duplicate using the MP Biomedical I125 RIA cortisol Kit (# 07-221106, MP Biomedicals Australia, Seven Hills, NSW). The limit of detection was 2.5 ng/mL and the mean inter-assay coefficients of variation were 3.6% (11.2 ng/mL) and 3.8% (53.5 ng/mL). Results are expressed as ng of FGCM/g of dry feces.

### Statistical analyses

Statistical analyses were performed in Stata (StataCorp, Release 16, College Station, TX). Kappa coefficients for the synchronization of lying were calculated for each 24-h period for each pen on days 2, 5, 7, and 9 (12 pens by 4 d = 48 kappa coefficients). Kappa coefficients were calculated using the numbers of steers lying at each of the hourly scan sampling time points taken at 19 time points within the 24-h periods, according to the methods outlined by Rook and Penning (1991). Briefly, kappa coefficients of agreement were calculated based on the concept of all possible pairs of steers that could be defined from the 5 steers, and the extent of synchronization at each time was determined by comparing the number of pairs where both steers were lying to the total number of possible pairs (Rook and Penning, 1991). The kappa coefficient was 1.0 for a pen for a 24-h period only if, at each time point within the 24-h period, all steers had the same lying status e.g., at some time points within the 24-h period, all steers were lying and at all other time points, all steers were standing. A kappa coefficient of 0.0 indicates that the number of steers lying within time points in the 24-h period was no greater than that expected by chance. Kappa coefficients were analyzed using multilevel linear regression models with pen-day as the unit of analysis, with pen included as a random effect, and with a first-order autoregressive residual correlation structure to account for correlations in residuals between days within pens. *k* value and day were simultaneously fitted as fixed effects, and as described above.

For counts of behaviors occurring in the pen at hourly timepoints, the unit of analysis was the hourly timepoint within the pen. For each standing or lying behavior (Table 1), mixed effects generalized linear model regression was used with pen included as a random effect, with Gaussian error distribution and log link function. Despite the small counts (range 0 to 5), regression models were considered to be suitable, in accordance with McDonald and White (2010). Diurnal patterns in counts were evident, so trigonometric (sine and cosine) predictors (Cox, 2006) with one complete period per 24-h were included to account for time-of-day effects. For all models except number standing, the number of steers eligible to exhibit the behavior was included in the model to account for the amount of ‘exposure’ (i.e., the number of animals for which the behaviors could have occurred and been observed). Thus, for analyses of number of lying steers that were in body contact with another steer, and analyses of the number of lying steers with legs outstretched from their body (lying positions 1, 2, 3, and 4 pooled; Table 1), the amount of exposure was the number of steers in the group that were lying at that time. For analyses of the number of lying steers placing their head on a conspecific (head position 4; Table 1) the amount of exposure was the number of steers lying with their head down. Exposure counts did not include steers for whom the behavior could not be observed on the image. Exponentiated coefficients from these models were interpreted as ratios of means. Other than for number standing, fitted means from regression models were plotted as approximate mean proportions of steers, by dividing fitted values from regression models by the average numbers of steers per pen included in exposure counts. Confidence intervals for these mean proportions were calculated using the delta method. For all 3 of these behaviors, within each of the 4 d they were sampled (days 2, 5, 7, and 9), residuals were positively correlated between adjoining timepoints for 2 to 8 of the 11 pairs of the immediately joining timepoints used in the models. However, there were no consistent correlations for more separated pairs of timepoints for any of the 3 behaviors on any sampling day. The correlation structures that explicitly account for autoregressive correlations between residuals for timepoints within pens are not available with Stata’s multilevel mixed-effects generalized linear model command. Given the lack of consistent correlations for most pairs of timepoints, we consider that the benefits of using a multilevel mixed-effects generalized linear model (specifically allowing use of the log link given the distribution of the data and the interpretability of the exponentiated coefficients from these models as ratios of means) outweigh the modest imperfection in accounting for residual correlations.

The number of aggressive interactions occurring in each pen during observation periods from 1030 to 1130 h on days 2, 5, 7, and 9 were analyzed using zero-inflated negative binomial models with robust standard errors that accounted for the clustering of observation period within pen. Pen-observation period was the unit of analysis.

To assess the effects of *k* value and day on liveweight, FGCM concentration, and blood variables, *k* value and day were simultaneously fitted as fixed effects in regression models. *k* value was fitted as a continuous variable, and day was fitted as a categorical variable. The day 0 value of the respective outcome variable was also fitted as a continuous variable, as a fixed effect. First, the linearity of any relationship between *k* value and the outcome variable was assessed using fractional polynomial regression. For all outcome variables, there was no strong evidence for nonlinear relationships for *k* value, so any relationship between *k* value and the outcome variables were assumed to be linear. Each of these outcome variables was first assessed separately on days 6 and 10. For all of these outcome variables, the estimated effects of *k* value on outcome variables were similar on each of these days, so the data were pooled and steer-day was the unit of analysis. Liveweight and blood variables were analyzed using multilevel linear regression models with pen and steer within pen were fitted as hierarchical random effects. FGCM concentration was analyzed using mixed effects generalized linear model regression with pen included as a random effect, with Gaussian family and with log link function. The use of models for the pooled data improved the standard error for the *k* value estimate in all cases. For FGCM concentration, exponentiated coefficients were interpretated as ratios of means.

For all outcome variables, terms for interaction terms between *k* value and day were jointly assessed using likelihood ratio test *P* values. Results for the interactions terms were only reported when the *P* value for the interaction terms was low. In interpreting our results, we implemented the principles listed in the American Statistical Association 2016 statement on statistical significance and *P* values (Wasserstein and Lazar, 2016) and the methods described by Goodman (2001) and Rothman and Lash (2021). Thus, we have considered our prior views about the probability that the null hypothesis is the truth in the target population when interpreting *P* values, as recommended by Goodman (2001), and considered the magnitude of alternative hypotheses that, based on our confidence intervals, are not compatible with our data and, assuming no prior information about the magnitude of effect, are unlikely to be the true value. As such, our description of effects as “important” or otherwise is not based on *P* values, but rather on the confidence interval limits in the context of real implications for cattle. In alignment with how the data has been interpreted, *P* values are only presented with the estimates and confidence intervals so that all information can be interpreted together, and *P* values are not presented on their own (i.e., in tables, figures, or elsewhere).

## Results

### Number of steers standing and lying

**Figure 2.**
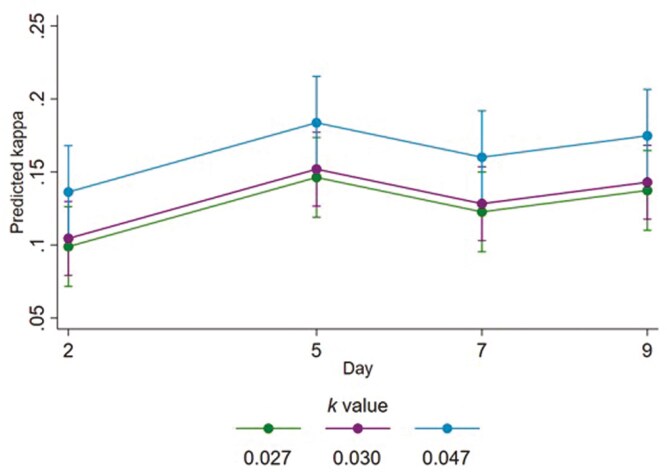
Fitted mean kappa coefficients for the synchronization of steers lying across behavior sampling days for each allometric stocking density coefficient (*k*). Error bars represent 95% confidence intervals of fitted means.

Kappa values for the synchrony of lying (calculated for each 24-h period for each group) ranged from 0.05 to 0.26. A small effect of *k* value was observed; for every 0.01 increase in *k* value (e.g., from *k* = 0.027 to *k* = 0.037), the mean kappa value for lying synchrony was estimated as increasing by 0.02 (95% CI 0.002 to 0.04; *P *= 0.029; Figure 2). Thus, the extent of synchrony increased by a small amount with more floor space per steer (i.e., with a higher *k* value). A small effect of the day was observed, in that relative to day 2, the mean kappa value for lying was estimated as being 0.05 (95% CI 0.02 to 0.08; *P *= 0.002), 0.02 (95% CI −0.01 to 0.06; *P *= 0.155), and 0.04 (95% CI 0.004 to 0.07; *P *= 0.025) greater on days 5, 7, and 9, respectively. Relative to day 5, mean kappa values were similar on day 7 (estimated difference 0.02; 95% CI −0.05 to 0.004; *P *= 0.092), and on day 9 (estimated difference 0.01; 95% CI −0.04 to 0.02; *P *= 0.583). Relative to day 7, mean kappa values on day 9 were also similar (estimated difference 0.01; 95% CI −0.01 to 0.04; *P *= 0.295).

**Figure 3.**
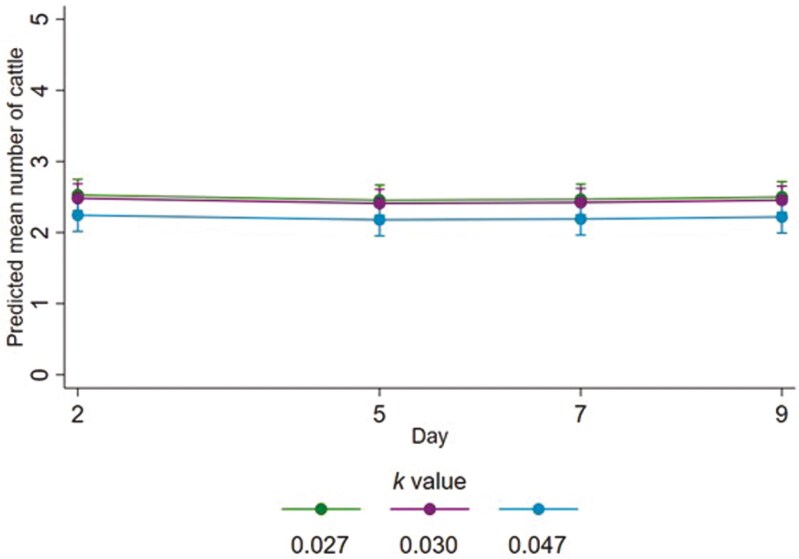
Fitted mean number of steers that were standing on each behavior sampling day, for each allometric stocking density coefficient (*k*). Error bars represent 95 % confidence intervals of fitted means.

The mean number of steers standing slightly decreased with more floor space per steer (i.e., with higher *k* value; Figure 3), estimated as decreasing by a factor of 0.94 for every 0.01 increase in *k* value (95% CI 0.90 to 0.99; *P *= 0.023). Estimated mean numbers of steers standing were similar on all behavior sampling days.

**Figure 4.**
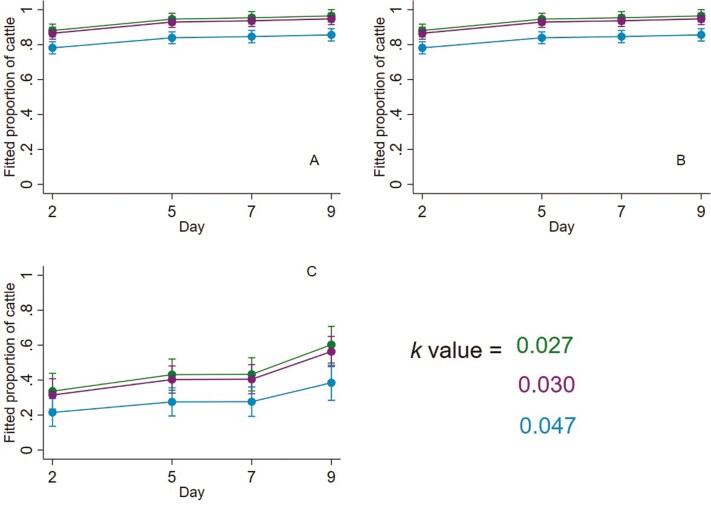
Fitted proportions of lying steers exhibiting specific lying behaviors on each behavior sampling day, for each allometric stocking density coefficient (*k*). Error bars represent 95 % confidence intervals of fitted proportions. (**A**) Fitted proportions of lying steers that were in physical body contact with a conspecific. Fitted proportions were calculated as fitted numbers divided by the average number of lying steers per pen whose body contact was known (3.29 steers). (**B**) Fitted proportions of lying steers that had any number of outstretched legs. The model included terms for interaction between the allometric stocking density coefficient (*k*) and day. Fitted proportions were calculated as fitted mean numbers divided by the average number of lying steers per pen whose leg position was known (3.25 steers). (**C**) Fitted proportions of lying steers that were resting their head on a conspecific. Fitted proportions were calculated as fitted mean numbers divided by the average number of lying steers per pen whose head position was known (1.54 steers).

The number of lying steers that were in contact with a conspecific decreased slightly with more available floor space, estimated as decreasing by a factor of 0.94 for every 0.01 increase in *k* value (95% CI 0.93 to 0.96; *P *< 0.001; Figure 4A). A small effect of the day was also observed, in that the number of lying steers that were in contact with a conspecific increased (relative to day 2) by a factor of 1.07 (95% CI 1.02 to 1.13; *P *= 0.005), 1.08 (95% CI 1.03 to 1.14; *P *= 0.002), and 1.10 (95% CI 1.04 to 1.15; *P *< 0.001) for days 5, 7, and 9 respectively. Relative to day 5, the number of lying steers in contact with a conspecific was similar on days 7 (1.01; 95% CI 0.96 to 1.06; *P *= 0.737) and 9 (1.02; 95% CI 0.97 to 1.07; *P *= 0.409). The number of lying steers in contact with a conspecific was similar on day 9 (1.01; 95% CI 0.96 to 1.06; *P *= 0.634), relative to day 7.

Of steers that were lying, there was evidence that the number lying with legs outstretched from their body (lying positions 1, 2, 3, and 4 pooled; Table 1) is determined, in part, by an interaction between *k* value and day (*P = *0.0287; Figure 4B). The number of lying steers with outstretched legs increased with increasing *k* value on days 2 and 5, but not on days 7 and 9. The estimated number of lying steers with outstretched legs increased by a factor of 1.09 (95% CI 1.02 to 1.18; *P* = 0.011) and 1.11 (95% CI 1.03 to 1.19, *P *= 0.004) for every 0.01 increase in *k* value on days 2 and 5, respectively. For days 7 and 9, for every 0.01 increase in *k* value, the numbers of lying steers with outstretched legs were estimated as increasing only by factors of 1.01 (95% CI 0.94 to 1.07; *P *= 0.845) and 1.01 (95% CI 0.95 to 1.07; *P *= 0.683), respectively.

*k* value had a small effect on the number of lying steers that were resting their head placed down (on the floor, themselves, or on a conspecific; Table 1). Of those lying, the number of steers with their head down was less with more space, estimated as decreasing by a factor of 0.87 for every 0.01 increase in *k* value (95% CI 0.79 to 0.97; *P *= 0.011). The number of lying steers with their head down also changed slightly across days. Relative to day 2, the number of lying steers with their head down was estimated as increasing by a factor of 1.10 (95 %CI 0.92 to 1.31; *P *= 0.308), 1.17 (95% CI 0.98 to 1.40; *P *= 0.076), and 1.32 (95% CI 1.11 to 1.56; *P *= 0.002) on days 5, 7, and 9, respectively. Relative to day 5, the number of lying steers with their head down was estimated as increasing by a factor of 1.07 on day 7 (95% CI 0.91 to 1.25; *P *= 0.406) and by a factor of 1.20 on day 9 (95% CI 1.03 to 1.39; *P *= 0.017). Relative to day 7, the number was estimated as increasing by a factor of 1.12 on day 9 (95% CI 0.97 to 1.30; *P *= 0.130).

Of steers that were lying with their head down, the number resting their head on a conspecific decreased as space increased. The number of steers resting their head on a conspecific was estimated as decreasing by a factor of 0.80 for every 0.01 increase in *k* value (95% CI 0.68 to 0.93; *P *= 0.005; Figure 4C). Some effect of day was also observed. Relative to day 2, the number of lying steers resting their head on a conspecific was estimated as increasing by a factor of 1.28 (95% CI 0.93 to 1.76; *P *= 0.128), 1.29 (95% CI 0.93 to 1.78; *P *= 0.128), and 1.80 (95% CI 1.33 to 2.40; *P *< 0.001) on days 5, 7, and 9, respectively. Relative to day 5, the number of lying steers resting their head on a conspecific was similar for day 7 (1.00; 95% CI 0.78 to 1.28; *P *= 0.973) but was estimated as increasing by a factor of 1.39 on day 9 (95% CI 1.14 to 1.71; *P *= 0.001). Relative to day 7, the number of lying steers resting their head on a conspecific on day 9 was estimated as increasing by a factor of 1.39 (95% CI 1.13 to 1.71; *P *= 0.002).

### Aggressive interactions

**Figure 5.**
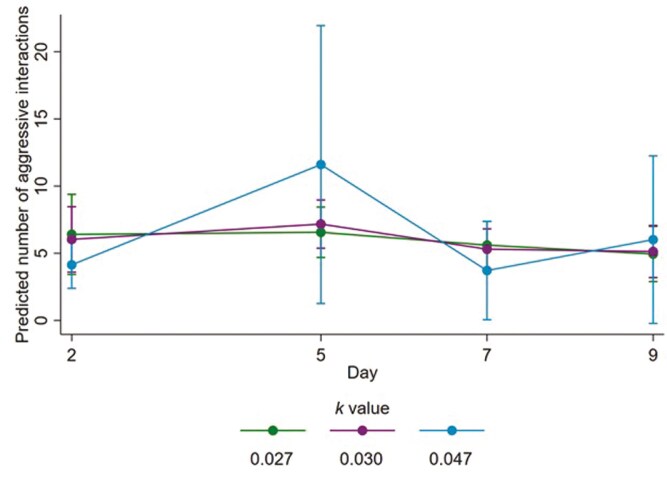
Fitted mean numbers of aggressive interactions from 1030 to 1130 h per pen for steers of each allometric stocking density coefficient (*k*) on each behavior sampling day. Error bars represent 95% confidence intervals of fitted means. A zero-inflated negative binomial model was used, and the fitted mean numbers account for the effects of *k* value and day in both the count and inflate components of the model.

For the number of aggressive interactions, considering the effects of the 2 parts of the zero-inflated model in combination, there were limited appreciable effects of stocking density on the counts of aggressive interactions. There was some evidence that steers housed at a *k* value of 0.047 (i.e., the lowest stocking density) performed a higher number of aggressive interactions on day 5 (Figure 5), but the wide confidence interval indicates the result is imprecise and so limited inferences can be drawn. Further exploration of the data indicated the high count of aggressive interactions observed for steers housed at a *k* value of 0.047 pens on day 5 was caused by only 1 of the 4 pens stocked at this density. The steers in this pen performed 139 aggressive social interactions in the day 5 observation period. The maximum number of aggressive social interactions in any other pen during the day 5 observation period was 56, and the mean number (±SD) of aggressive interactions across all pens and all periods was 29.8 ± 23.0.

### Fecal glucocorticoid metabolite concentrations

**Figure 6.**
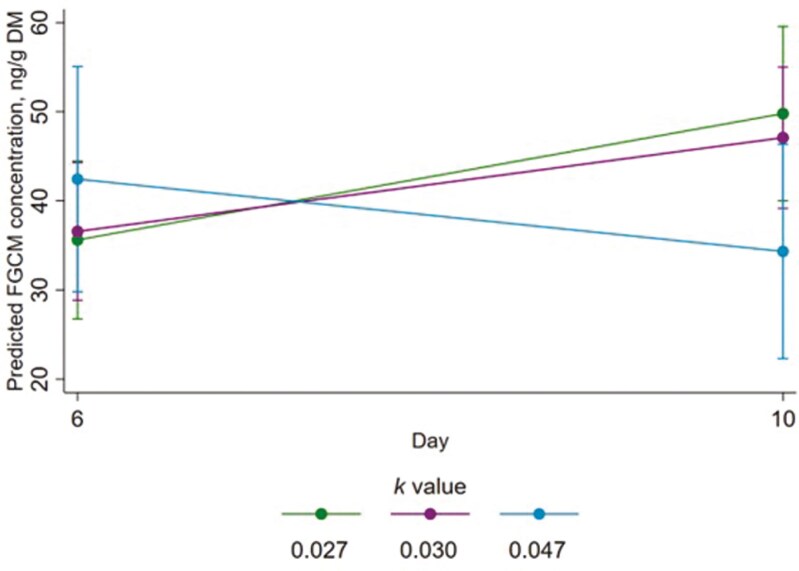
Fitted FGCM concentrations of focal steers for the interaction between day and allometric stocking density coefficient (*k*). Error bars represent 95% confidence intervals of fitted means.

The baseline concentrations of FGCM concentrations, and the change in concentration on days 6 and 10 relative to the baseline, are shown for each stocking density in Table 2. There was evidence of interaction between *k* value and day for fecal glucocorticoid metabolite concentrations (*P* = 0.037), but the magnitude of the estimated interaction was modest (Figure 6). On day 6, there was no important effect of *k* value on fecal glucocorticoid metabolites; for every 0.01 increase in *k* value, FGCM concentrations were estimated as increasing by a factor of 1.09 (95% CI 0.89 to 1.34; *P* = 0.402). On day 10, there was no important effect of *k* value on fecal glucocorticoid metabolites; for every 0.01 increase in *k* value, FGCM concentrations were estimated as decreasing by a factor of 0.83 (95% CI 0.67 to 1.03; *P* = 0.085).

**Table 2.** Mean ± SD day 0 FGCM concentrations (ng/g DM) for focal steers and the means ± SD of the changes (Δ) within steer at each sampling day relative to day 0 (ng/g DM) for each allometric stocking density coefficient (*k*)

*k* value	Day 0	Δ6	Δ10
0.027	54.03 (±19.94)	14.90 (±26.29)	−0.65 (±25.70)
0.030	63.42 (±28.10)	27.81 (±24.05)	21.81 (±34.16)
0.047	46.77 (±19.45)	6.08 (±20.91)	12.12 (±24.42)

### White blood cell counts

White blood cell counts are summarized in Table 3. All mean counts fell within accepted reference intervals for healthy cattle, but a small proportion of individual animals had counts either below or above the accepted reference intervals.

**Table 3.** Day 6 and 10 total white blood cell, lymphocyte, and neutrophil counts (× 10^6^ cells/mL) and day 6 and 10 neutrophil to lymphocyte ratio expressed as means ± SD, for focal steers housed at each allometric stocking density coefficient (*k*), and the proportion of total steers (*n* = 35) with values lower (low) or higher (high) than the reference interval for each white blood cell count and the neutrophil to lymphocyte ratio on day 6 and 10

Variable	Day 6					Day 10					Reference interval
	0.027	0.030	0.047	Low	High	0.027	0.030	0.047	Low	High	
White blood cell count, × 10^9^ cells/L	10.5 ± 2.2	9.8 ± 1.8	10.1 ± 2.2	0.00	0.17	10.5 ± 2.2	9.3 ± 1.3	9.2 ± 1.3	0.00	0.11	4.9 to 12.0^1^
Lymphocyte count, × 10^9^ cells/L	6.2 ± 1.6	5.9 ± 1.3	5.6 ± 0.9	0.00	0.42	5.0 ± 1.8	4.9 ± 1.1	4.8 ± 1.3	0.00	0.29	1.6 to 5.6^1^
Neutrophil count, × 10^9^ cells/L	3.5 ± 1.1	3.0 ± 1.1	3.9 ± 1.8	0.09	0.03	4.7 ± 2.3	3.7 ± 0.7	3.9 ± 1.0	0.03	0.06	1.8 to 6.3^1^
Neutrophil to lymphocyte ratio	0.6 ± 0.2	0.5 ± 0.3	0.7 ± 0.3	0.23	0.00	1.1 ± 0.8	0.8 ± 0.3	0.9 ± 0.6	0.14	0.06	0.4 to 2.34^1^

^1^Reference intervals as reported by George et al., 2010.

From these results, any effects of stocking density on total white blood cell counts, lymphocyte counts, and neutrophil counts are small. For total white blood cell counts, the estimated effect was an increase of 0.10 × 10^6^ cells/ml (95 % CI −0.35 to 0.55 × 10^6^ cells/mL) for every 0.01 increase in *k* value (i.e., more space; *P* = 0.654). A small effect of the day was observed, in that total white blood cell counts were estimated to decrease by 0.50 × 10^6^ cells/ ml between day 6 and 10 (95% CI −0.89 to −0.10 × 10^6^ cells/mL; *P *= 0.013).

For lymphocyte counts, the estimated effect was a decrease of 0.09 × 10^6^ cells/mL (95% CI −0.41 to 0.24 × 10^6^ cells/mL) for every 0.01 increase in *k* value (i.e., more space; *P* = 0.595). A small effect of day was observed, in that lymphocyte counts were estimated to decrease by 1.00 × 10^6^ cells/ml between day 6 and 10 (95% CI −1.39 to −0.61 × 10^6^ cells/mL; *P *< 0.001).

For neutrophil counts, the estimated effect was an increase of 0.17 × 10^6^ cells/mL (95% CI −0.20 to 0.54 × 10^6^ cells/mL) for every 0.01 increase in *k* value (i.e., more space; *P* = 0.375). A small effect of day was observed, in that neutrophil counts were estimated to increase by 0.65 × 10^6^ cells/mL between day 6 and 10 (95% CI 0.10 to 1.20 × 10^6^ cells/mL; *P *= 0.021).

Furthermore, there were no important effects of *k* value on the neutrophil-to-lymphocyte ratio. For every 0.01 increase in *k* value, the ratio was estimated as increasing by a factor of 1.04 (95% CI 0.87 to 1.21, *P* = 0.673). A small effect of day was observed, in that the neutrophil to lymphocyte ratio was estimated as increasing by a factor of 1.75 between days 6 and 10 (95% CI 1.42 to 2.16; *P *< 0.001).

### Liveweights

Mean (± SD) day 0, 6, and 10 liveweights for cattle housed at *k* value stocking densities of 0.027, 0.030, and 0.047 are shown in Table 4. There was no important effect of *k* value (estimated change in mean for every 0.01 increase in *k* value = 0.33 kg; *P *= 0.755) on liveweight on days 6 and 10 collectively. However, the 95 % CI (−1.73 to 2.39) suggests the data are compatible with possible small effects of *k* value on the liveweight of cattle at either time point. The estimated difference in mean between days 6 and 10 = 1.6 kg (95 % CI −0.34 to 3.45; *P* = 0.108).

**Table 4.** Day 0, 6, and 10 steer liveweights (mean kg ± SD) for each allometric stocking density coefficient (*k*)

*k* value	Day 0	Day 6	Day 10
0.027	309.5 (±19.4)	306.6 (±18.6)	309.9 (±20.1)
0.030	304.3 (±20.7)	305.0 (±22.3)	306.3 (±24.7)
0.047	315.7 (±23.9)	315.5 (±23.4)	315.5 (±23.8)

## Discussion

This study was conducted to assess selected measures that reflect the welfare of cattle housed at 3 allometric *k* value stocking densities, as indicated by measures of behavior, glucocorticoid hormone metabolites, white blood cell counts, and liveweight. Some effects of *k* value on cattle behavior were observed, in that reduced pen space led to a small reduction in both the number of steers lying and synchrony of lying. In addition, when provided with additional pen space, fewer steers chose to lay in physical body or head contact with a conspecific. The lack of important effects of stocking density on FGCM concentrations, liveweights, and white blood cell counts, for the 10-d period of the current study, suggests that the pen space restriction did not contribute to changes in these physiological measures, despite the behavioral effects observed (i.e., the cost of coping was relatively low, manifested by only behavioral responses). However, the apparent absence of large effects on the physiological and biological fitness variables may also have been due to the short duration of the study, and further impacts on these variables may occur for periods of time greater than 10 d. While this study was designed to be applicable to Australian livestock export voyages, other factors which might impact cattle welfare in this environment were not present. The treatments included in the study were limited to stocking densities alone, to gain an understanding of the role of this factor in isolation. As such, future work will benefit from increasing the complexity of the environment, to provide information on the impact of other relevant factors, as well as their potential interactions with stocking density.

Deciding which behaviors are necessary for life, health and comfort is difficult (Hurnik and Lewis, 1991), but a free opportunity to lie down is considered a basic requirement of ruminants, and lying is a useful metric of animal behavior with regard to welfare (Phillips and Petherick, 2015; Temple et al., 2016). Lying is particularly important for cattle, which have been found to sacrifice feeding time to obtain additional resting opportunities, after both feeding and lying have been restricted (Metz, 1985). In the current study, the provision of additional pen space led to a very small reduction in the number of steers standing (i.e., an increase in the number of steers lying). In addition, more pen space also led to a slight increase in synchronous lying. Cattle are a gregarious species, so behaviors such as lying down, eating, and drinking are often synchronized (Miller and Wood-Gush, 1991), but this synchrony may breakdown in intensive housing environments if there is competition for resources (e.g., competition for lying space; Miller and Wood-Gush, 1991). It has been suggested that a *k* value of 0.027 (i.e., the highest stocking density in the current study) is sufficient for synchronous lying in ruminant animals (Petherick and Phillips, 2009). Previous work has indicated this is the case for both sheep (Mayes et al., 2022) and cattle (Fregonesi and Leaver, 2002), and while *k* value did have some effect on the synchrony of lying for cattle in the current study, the estimated increase in synchrony was small (0.02 increase in kappa for every 0.01 increase in *k* value, i.e., *k* = 0.027 to 0.037), and is likely trivial in its potential to impact overall resting ability or welfare. In addition, the increase in the number of steers lying with additional pen space was also small and unlikely to have any important effect on the ability of cattle to achieve sufficient rest.

For lying synchrony, an effect of day was also observed, and synchronicity of lying increased on days 5 and 9, relative to day 2. This may reflect the steers adapting to their new social group, as social cohesion is likely to impact the synchrony of behaviors, including lying (Plesch et al., 2010; Marino and Merskin, 2019). This is particularly relevant in the context of the stocking densities assessed, as synchronous lying could only be achieved if some, or potentially even all 5 steers within a pen, lay in physical contact with a conspecific. The amount of synchronous lying on day 7 was similar to that on day 2 (i.e., lower than for days 5 and 9), and may reflect the steers readapting to the pen environment after having been removed for several hours on day 6 for the collection of physiological samples. The increase in synchronicity of lying on day 5 relative to day 2, is particularly important in the context of short-haul livestock export voyages and suggests that cattle housed at the stocking densities investigated here may be able to adapt to their pen and social environment and achieve normal lying behaviors within only a few days. Other work has also found that normal lying times are generally achieved within the first few days of a livestock export voyage (Ferguson and Lea, 2013).

As well as these minor effects on the number of lying steers and the synchrony of lying, stocking density also affected the lying positions adopted by the steers. More pen space resulted in slightly fewer steers lying in body contact with conspecifics, and fewer steers resting their head on a conspecific. These results suggest that cattle may have a preference to lie in physical isolation from conspecifics when their pen space permits them to do so. Research conducted with both ewes (Bøe et al., 2006) and wethers (Mayes et al., 2022) has also indicated these classes of animals display a preference for lying in physical isolation when more lying space is available. In the current study, some effects of day on lying postures were also observed. Compared to day 2, the proportion of steers lying in body contact with conspecifics was higher on all other days, potentially indicating adaptation to their new environment and social group (Metz and Mekking, 1984).

In the current study, there was also an interaction between *k* value and day for the number of lying steers that had outstretched legs. Increased pen space resulted in more of the lying steers having outstretched legs on days 2 and 5, but not on days 7 and 9. Research on buffalo housed with space allowance provisions corresponding to either 50% or 90% of their estimated body surface area also showed that low pen space allowance reduced the number of outstretched legs observed in lying animals (Napolitano et al., 2004). The proportion of steers lying with outstretched legs also generally increased over time and was similar for all stocking densities on days 7 and 9, suggesting all pen space allowances in the study were sufficient in facilitating lying with some outstretched legs. Lying positions that are more comfortable, such as lateral recumbency with more legs outstretched, or sternal recumbency with the head tucked back near the flank, may indicate reduced vigilance or thermal comfort in cattle (Napolitano et al., 2009). These positions are more likely to be adopted if the animal feels its environment is predictable (Napolitano et al., 2009). As such, the reduced number of steers lying with outstretched legs at the start of the study for higher stocking density pens may indicate the additional time required to adapt to their pen environment and new social group. Whether being unable to lie in preferred positions (i.e., in physical isolation, or with outstretched legs) has further impacts on cattle welfare remains unclear and requires further research. Of note, while the current study was undertaken in thermoneutral conditions, it is important to consider that animals exposed to warm conditions during sea transportation may be further impacted by stocking density, with regards to resting in physical isolation and with body more spread out (i.e., more legs outstretched from the body; Herbut et al., 2021), due to the role of body surface area in heat dissipation and exchange between individuals in close contact (Knowles et al., 1998; MAMIC, 2001).

Stocking density had no important effect on several measures included in the current study—the counts of aggressive interactions, FGCM concentrations, and counts of total white blood cells, lymphocytes, neutrophils, or the neutrophil to lymphocyte ratio. We anticipated that increasing stocking density would result in increased counts of aggressive interactions, as similar results have been observed in buffalo calves (Napolitano et al., 2004) and dairy cows (Metz and Mekking, 1984; Fregonesi and Leaver, 2002). One explanation for the lack of effect of stocking density may be that the steers experienced a similar level of competition for lying space across all treatments. Displacements and aggression may be increased if animals perceive competition to access a valuable resource, including lying space (Richmond et al., 2017). The stocking densities investigated in the current study were relatively similar, and so limited effects of stocking density on the number and synchronicity of steers lying indicates a similar level of competition for lying space among all treatment groups. The lack of effect of stocking density on aggressive behaviors may have also partly resulted from the small group size of 5 steers per pen, utilized in the study. As group size increases, so too does competition for resources (Leme et al., 2013). Future work would benefit from exploring the effects of these stocking densities for larger groups of cattle. FGCM concentrations were also largely unaffected by stocking density for the duration of the current study, and previous stocking density research has also reported a lack of effect of pen space allowance on HPA axis activity (Fisher et al., 1997; Napolitano et al., 2004; Navarro et al., 2018). Stocking density also had no important effect on the counts of total white blood cells, lymphocytes, neutrophils, or the neutrophil-to-lymphocyte ratio in the current study, which is a similar outcome observed in previous stocking density research conducted for steers (Hickey et al., 2003) and sheep (Mayes et al., 2022). In addition, all values on days 6 and 10 fell within reference intervals for healthy cattle. Effects of stress on total white blood cell, lymphocyte, and neutrophil counts in ruminants have been documented previously (Hulbert et al., 2011; Pascual-Alonso et al., 2017), but these changes may arise through increased glucocorticoid concentrations (not observed in the current study) altering the numbers and distribution of white blood cell types in the blood and other body tissues (Davis et al., 2008; Trevisi and Bertoni, 2009).

Some differences in white blood cell counts between days 6 and 10 were observed, irrespective of the stocking density treatments. Counts of total white blood cells and lymphocytes decreased between days 6 and 10, while counts of neutrophils increased between days 6 and 10. A reduction in total white blood cells may indicate immunosuppression associated with stress, while an increase in the neutrophil-to-lymphocyte ratio may also indicate stress exposure (which induces an increase in neutrophil concentrations; Dhabhar, 2014). Neutrophil and lymphocyte counts are affected by stress in opposite directions, so the ratio of one to the other is considered by researchers to be a useful composite measure of stress responses (Davis et al., 2008). The change in white blood cell counts between days 6 and 10 implies that if a voyage is 10 d (i.e., long haul voyage) rather than 6 d (i.e., short-haul voyage), there may be further consequences for animal health and disease risk. Further research, which includes additional measures, is needed to better understand the changes in white blood cell counts observed between days 6 and 10 and their potential implications for animal health during longer voyages. It is unclear whether this trend would continue beyond day 10, especially given the lack of an important increase in FGCM concentration over time, which is a key mechanism through which stress affects white blood cell counts (Davis et al., 2008; Trevisi and Bertoni, 2009).

The lack of an important effect of *k* value stocking density on liveweight suggests that despite the behavioral effects observed, the increases in stocking density were not stressful enough to reduce liveweights (an important aspect of biological fitness; Broom, 1991). From voyage-based research, there was also no evidence that liveweight and liveweight gain was affected by stocking density (Ferguson and Lea, 2013). In the current study, there was evidence that small increases in liveweight (up to 2.4 kg for every 0.01 increase in *k* value) are compatible with the data, suggesting there may be slight improvements to cattle productivity if additional pen space is provided. Effects of stocking density on the liveweight of cattle have been previously reported, particularly with regard to the relationships between physiology or behavioral changes and liveweight (Fisher et al., 1997). Animals that were provided with the least amount of space per head (1.5 m^2^, compared to 2.0, 2.5, or 3.0 m^2^, or *k* ≈ 0.026, 0.035, 0.043, or 0.052, respectively) displayed less time spent lying and time spent ruminating (Fisher et al., 1997). As well as these behavioral results, the cattle with the least space had reduced average daily gain and final bodyweight, when compared to all other treatments (Fisher et al., 1997). Mean times spent feeding were similar across all stocking density treatments, so the authors suggested the observed liveweight reductions may have resulted from an effect of reduced space on the efficiency of feed conversion (i.e., energy for growth was diverted to account for the increases in time spent standing; Fisher et al., 1997). The current study investigated a similarly high stocking density but did not see that higher stocking densities lead to reductions in liveweight, likely because numbers of steers lying were only slightly reduced at higher stocking densities. Where limited physiological effects of stocking density have been observed in cows, this has been partially attributed to the ability of cows to adapt to a competitive resting environment (Huzzey et al., 2012), and this adaptive ability may also explain the changes in lying behaviors observed throughout the current study, specifically, increases in lying synchrony and proportion of steers lying with outstretched legs over time.

Appropriate interpretation of results from this study relies on the consideration of several important factors. Firstly, the range of stocking densities investigated in the current study is relatively narrow, to maintain relevance to Australia’s livestock export industry. The provision of more or less pen space outside the range of stocking densities investigated may impair or improve cattle welfare in ways which were not identified. In addition, it is critical to consider that the treatment stocking densities were imposed for a period of 10 d, to simulate short-haul voyages (Department of Agriculture, Fisheries and Forestry, 2024) lasting 6 and 10 d, and cattle may experience additional stress relative to each stocking density if housed under these conditions for longer periods of time (i.e., for long- or extended long-haul voyages). Directly investigating the effect of stocking density on cattle welfare during long- or extended long-haul voyages was outside the scope of the current study, and future research may benefit from investigating these longer timeframes. As part of this, it would be important for researchers to account for the potential effects of changes in weather that may be experienced over longer distances, as well as any differences in the breed and class of cattle that are exported to the relevant destination countries. Additional stress may also result if additional stressors, not included in the current study, are present in the environment. Some other factors that may induce stress during Australian livestock export voyages include feed resource competition, fecal pad conditions, human activity, movement associated with ocean swell, and hot and humid weather. Future work should aim to investigate how stocking density may interact with other factors to impact cattle welfare in this context. For practical reasons, the size of the treatment pens in the current study, and thus the number of steers that were housed in each group, was limited. This meant that a dominance hierarchy was likely to be more rapidly established compared to if the group was larger. It also meant that distances to other resources (i.e., feed) were short compared to what would typically be experienced during a commercial livestock export voyage (Phillips and Petherick, 2015). As such, there may be further consequences of each stocking density tested for larger groups of steers that were not able to be identified here. In addition, the calmest steers (based on the crush score) were purposefully included in this research, which may limit the applicability of the results to more diverse groups which include steers that are less calm.

## Conclusions

The aim of this study was to assess some indicators of welfare for cattle housed at 3 allometric stocking densities. The measures included cattle behavior, fecal glucocorticoid metabolite concentrations, white blood cell counts, and liveweight (i.e., indicators of biological functioning and fitness). Stocking density had an effect on the frequency of some lying positions, suggesting that cattle were unable to lie in preferred positions and that they took longer to adapt to their environment when provided with less pen space (i.e., at low *k* values). We also provide evidence that pen space restriction did not contribute to physiological stress or impaired biological fitness, indicating that the biological cost of coping was relatively low and achieved with behavioral responses only—at least for the 6- and 10-d periods investigated here. This research has provided valuable insight into the importance of stocking density for cattle which are housed intensively or exported by sea, but the conclusions must be interpreted in the context of the highly controlled experimental environment. Future research should aim to determine the cumulative effects of other important factors that may impact cattle during livestock export voyages (e.g., wave motion, heat, and humidity), and how such factors may also interact with pen space allowance.

## Supplementary Material

skaf034_suppl_Supplementary_Materials

## Abbreviations

CI: confidence interval
CP: crude protein
DM: dry matter
FGCM: fecal glucocorticoid metabolite
HPA: hypothalamic-pituitary-adrenal
LW: liveweight
ME: metabolisable energy
QASP: Queensland animal science precinct
RH: relative humidity
SE Asia: South-East Asia
T_DB_: dry-bulb temperature
T_WB_: wet-bulb temperature
