# Supplementary material for: Stocking density in intensive housing and the implications for beef cattle behavior, stress physiology, and liveweight
Source: J Anim Sci. 2025 Feb 7;103:skaf034. doi: 10.1093/jas/skaf034 (PMC11926537; doi:10.1093/jas/skaf034)
Supplement: skaf034_suppl_Supplementary_Materials [file skaf034_suppl_supplementary_materials.docx]

**Supplementary Materials**

Supplementary Table 1 Description used to assess the temperament of each steer for 30 s, upon entry into the crush.

| **Crush score** | **Description of cattle behaviour** |
| --- | --- |
| 1 | Calm, standing still, head mostly still, slow calm movements |
| 2 | Slightly restless, looking around more quickly, moving feet, shifting weight |
| 3 | Restless, moving backward and forward, some slight movement of crush |
| 4 | Nervous, continuous vigorous movement backward and forward, snorting, some movement of crush |
| 5 | Very nervous, violent movements, rearing, attempting to jump out |

**Supplementary Table 2** Mean daily dry-bulb temperature (T_DB_), relative humidity (RH), and wet-bulb temperature (T_WB_) over the 10-day study period.

| **Day** | **T_DB,_ °C** | **RH, %** | **T_WB,_ °C** |
| --- | --- | --- | --- |
| 0 | 19.6 | 75.5 | 16.7 |
| 1 | 20.9 | 77.8 | 18.2 |
| 2 | 21.5 | 81.0 | 19.1 |
| 3 | 22.1 | 78.5 | 19.4 |
| 4 | 21.9 | 79.8 | 19.4 |
| 5 | 20.3 | 81.6 | 18.2 |
| 6 | 20.1 | 72.6 | 16.7 |
| 7 | 19.5 | 71.7 | 16.1 |
| 8 | 20.5 | 74.8 | 17.4 |
| 9 | 22.5 | 76.7 | 19.5 |
| 10 | 21.9 | 86.2 | 19.9 |

**Supplementary Table 3** Coefficients for Lin’s Concordance correlation (LCC) and intra-class correlation (ICC) between the trainer (BM) and observer (SD) for scan sampling behaviours. Agreement results are based on 76 observation timepoint images.

| **Behaviour** | **LCC** | **ICC** |
| --- | --- | --- |
| Standing | 0.998 | 0.997 |
| Lying 0 | 0.805 | 0.802 |
| Lying 1 | 0.884 | 0.834 |
| Lying 2 | 0.741 | 0.822 |
| Lying 3 |  |  |
| Lying 4 |  |  |
| Body 1 | 0.968 | 0.956 |
| Body 2 | 0.758 | 0.747 |
| Head 1 | 0.900 | 0.871 |
| Head 2 | 0.429 | 0.657 |
| Head 3 | 0.885 | 0.900 |
| Head 4 | 0.500 | 0.735 |
